# Expression and localization of maspin in cervical cancer and its role in tumor progression and lymphangiogenesis

**DOI:** 10.1007/s00404-013-2988-4

**Published:** 2013-08-20

**Authors:** Zhiqiang Liu, Yangyang Shi, Wei Meng, Yufang Liu, Kaixuan Yang, Shuhua Wu, Zhilan Peng

**Affiliations:** 1 Department of Obstetrics and Gynecology, Affiliated Hospital of Binzhou Medical College, Binzhou, 256603 China; 2 Department of Obstetrics and Gynecology, Peking University First Hospital, Beijing, 100034 China; 3 Laboratory of Pathogenic Organism, Affiliated Hospital of Binzhou Medical College, Binzhou, 256603 China; 4 Department of Pathology, West China Second Hospital (Women and Infants Hospital) of Sichuan University, Chengdu, Sichuan 610041 China; 5 Department of Pathology, Affiliated Hospital of Binzhou Medical College, Binzhou, 256603 China; 6 Department of Obstetrics and Gynecology, West China Second Hospital (Women and Infants Hospital) of Sichuan University, No.17, Renmin Road South, Chengdu, Sichuan 610041 China

**Keywords:** Cervix, CIN, Maspin, Lymphatic system, Podoplanin, Squamous cell carcinoma

## Abstract

**Objectives:**

Cervical cancer is the most common malignant tumor in female reproductive tract and primarily metastasizes through the lymphatic system that will affect prognosis of patients. Maspin, a member of the serine protease inhibitors (serpins) super family, has recently been indicated as a tumor suppressor in many cancers. In this study, we investigated the clinical significance of maspin expression, especially the subcellular location of maspin and its functional role in progression and lymphangiogenesis, in cervical squamous cell carcinoma.

**Methods:**

Labelled streptavidin biotin method (LSAB) was used to determine cytoplasmic and nuclear maspin expressions, respectively, in 13 cases of normal cervix, 15 cases of cervical intraepithelial neoplasia grade 3 (CIN3), 62 cases of squamous cell carcinoma (SCC) of the uterine cervix, and 13 cases of pelvic lymphatic nodes which were all positive lymphatic nodes in our selected cancer cases. LSAB is also used to detect podoplanin which is used for counting density of lymphatic microvessels (LMVD). The clinical significance of subcellular maspin expression and the relationship between maspin expression and LMVD in cervical cancer are analyzed.

**Results:**

Both cytoplasmic and nuclear maspin expressions in SCC were significantly weaker than those of normal cervix and CIN3. Nuclear maspin expression showed a peak in CIN3 and then dropped in SCC. Declined maspin expression was correlated with later clinical stage, increased LMVD, and lymphatic metastasis.

**Conclusions:**

Our results suggest that subcellular location of maspin expression is a potential predictive factor in tumor progression and in patients’ prognosis of cervical cancer, and maspin plays a suppression role in lymphangiogenesis and metastasis.

## Introduction

Maspin is a serine protease inhibitor located at 18q21.3, along with other serpin genes such as squamous cell carcinoma antigens 1 and 2, and PAI-2 [1]. It was identified in 1994 as a tumor suppressor gene from normal mammary epithelial cells [2]. Since then, accumulated evidence has demonstrated its tumor-suppression role [2–7], which was further enhanced by recent studies that showed downregulation or loss of maspin expression indicated greater propensity for metastasis and poor prognosis in patients, whereas upregulation of maspin is one of the independent factors for good prognosis [6–9]. Maspin inhibits cell growth and motility, weakens the potential for invasion and metastasis [8, 10], and engenders a sensitizing effect on apoptosis [11]. Nevertheless, despite its declined expression in many kinds of cancers, maspin was overexpressed in some other tumors, such as colonic carcinoma, ovarian carcinoma, and gallbladder cancer [12–15]. New evidence showed that maspin expression in breast cancer was positively related to tumor size and histological grade, but negatively related to relapse-free survival and total survival spans of patients, which questioned maspin’s role as a tumor suppressor [10, 16–18]. The paradoxical expressions of maspin in different types of tumors may provide new insights regarding the role of maspin in tumor progression, metastasis and prognosis. Furthermore, a few recent studies showed that cytoplasmic and nuclear maspin expressions may predict the different clinical types in prognosis of patients in ovarian cancer, non-small-cell lung cancer and laryngocarcinoma [2, 19–21], which suggested that even further significance may exist in subcellular localization of maspin.

Carcinoma of the uterine cervix is one of the most common malignancies in female genital tract in which lymphatic metastasis is the primary route of spread out and a cardinal factor for prognosis of patients. Podoplanin is one of the complete membrane-spanning proteins with molecular weight 43 Kd that was first discovered in glomerular epithelial cells in 1999 [22] and had been proved to be an ideal specific marker for endothelial cells of lymphatic vessels [23].

At present, the molecular and biological mechanisms of maspin functions are still unclear. Only a few studies have been performed to determine the maspin expression and its relationship with angiogenesis and vascular metastasis in cervical cancer, whereas subcellular location of maspin expression and its association with lymphangiogenesis and lymphatic metastasis remains blank. In our study, we evaluate cytoplasmic and nuclear maspin expression respectively in normal cervix, cervical intraepithelial neoplasia grade 3 (CIN3) and squamous cell carcinoma (SCC) of the uterine cervix and the density of lymphatic microvessels (LMVD) in the tumor tissue to discover the relationship between maspin subcellular expression and progression as well as lymphangiogenesis and lymphatic metastasis in cervical cancer.

## Methods

### Tissue samples

We conducted a retrospective study of maspin expression. A total of 13 cases of normal cervix which were all leiomyoma of the uterus, 15 cases of CIN3, 62 cases of SCC of the uterine cervix, and 13 cases of pelvic lymphatic nodes, which were all positive lymphatic nodes in our selected cancer cases from the period October, 2004 to April, 2006 were retrieved from the archival files of the Department of Pathology, West China Second Hospital of Sichuan University. No patient of cervical cancer has received radiotherapy, chemotherapy and biotherapy preoperatively. The pathologic diagnosis was definitely postoperative, which was reviewed by two experienced pathologists, and both the clinical and the pathologic data were complete. The clinical stage of cervical cancer was defined according to the criteria set forth by International Federal of Obstetrics and Gynecology (FIGO) in 2000. The whole study received permission from the ethics committees of China.

### Immunohistochemistry methods

Both maspin and podoplanin expressions were detected by labelled streptavidin biotin method (LSAB). Formalin-fixed, paraffin-embedded tissue blocks were sectioned at 4 μm intervals in four pieces, which were mounted on glasses with APES prime coat, baked under 60 °C for 3 h and turned to constant temperature in an oven at 37 °C for 48 h. Deparaffinized in xylol third for 10 min each, and hydrated gradually through graded alcohols (100 %, 95 %, 80 % and 70 % ethanol each for once). To enhance the immunostaining, an antigen retrieval procedure was performed. The deparaffinized maspin sections were placed in an autoclave sterilizer filled with 0.01 M citrate buffer solution pH 6.0, steamed for 4 min, and then washed with PBS (pH 7.2) third after natural cooling. The podoplanin sections were placed in a microwave oven in high gear filled with 0.01 M citrate buffer solution pH 6.0 for 5 min and then in middle gear for 10 min and washed with PBS (pH 7.2) third for 5 min each after natural cooling under room temperature. The sections were sequentially incubated in goat blocking serum in PBS, 1:500 dilutions of monoclonal maspin antibody (NeoMarkers, CA, USA), or 1:100 dilutions of polyclonal podoplanin antibody (AbD Serotec, UK), biotinylated secondary antibody IgG, and avidin-biotinylated peroxidase. After a final wash with PBS, chromogenic detection and hematoxylin counterstain were performed.

Positive and negative controls were set. Maspin expression took normal breast tissue and podoplanin took lymphatic nodes as positive controls while two first antibodies replaced with PBS respectively were taken as negative controls.

### Results determination

Characteristic cytoplasmic and nuclear granular staining of yellow or yellow brown is considered positive and the immunoreactivity was scored using a scale of 0 to 3 based on the percentage of positive cells, intensity of staining and heterogeneity of staining [1].

Two pathologists (KX Y, SH W) reviewed the slides independently to evaluate the maspin expression. Four high-power fields (×400) were chosen in each slide to evaluate cytoplasmic and nuclear maspin expressions, respectively.

For assessing LMVD, the reports of Weidner [24] and Jackson [25] were consulted for vessel counting. First, scan the whole section under low-power field (×40) and choose four fields of high-density area of lymphatic microvessels called “hot spot” to count the number under high-power field (×400). The mean number of lymphatic microvessels of the four fields was taken as the result. Vessels which contained more than eight endothelial cells or smooth muscles were not counted.

### Statistical analysis

The sums of scores from different test groups of maspin expression were statistically compared using the Kruskal–Wallis rank sum test. Maspin expression, density of LMVD and clinical or pathologic parameters were compared using the logistic regression analysis while the correlation among the group of SCC. All patients of SCC were free of them in SCC were analyzed using the Spearman rank correlation analysis, *t* test and Mann–Whitney rank test.

## Results

The range and mean age of patients in each group is shown in (Table 1). There are 49 premenopausal and 13 postmenopausal patients, respectively, in the SCC group. All patients of SCC were free of radiotherapy and chemotherapy, preoperatively.

**Table 1:** Range and mean age of patients of different study groups

Diagnosis	Age range (years)	Mean age (years)	Case number
Normal cervix (myoma of the uterus)	33–52	39.78 ± 8.56	13
CIN3	28–55	40.56 ± 7.26	15
SCC	27–74	42.78 ± 12.64	62
SCC with lymphatic nodes metastasis	34–62	44.37 ± 9.52	13

*SCC* Squamous cell carcinoma

Among 62 patients of SCC, there are 15 stage Ib and 47 stage II. For histological grade, 17 patients were high to moderate differentiation and 45 were poor differentiation. There were 13 cases of local lymphatic metastasis, and the other 49 cases were free of it. Invasive depth of interstitial and muscular layer is less than 1/2 in 14 cases and more or equal to 1/2 in 48 cases according to the pathologic reports, postoperatively.

### Maspin expression and subcellular localization in normal cervix, CIN3 and SCC

**Fig. 1:**
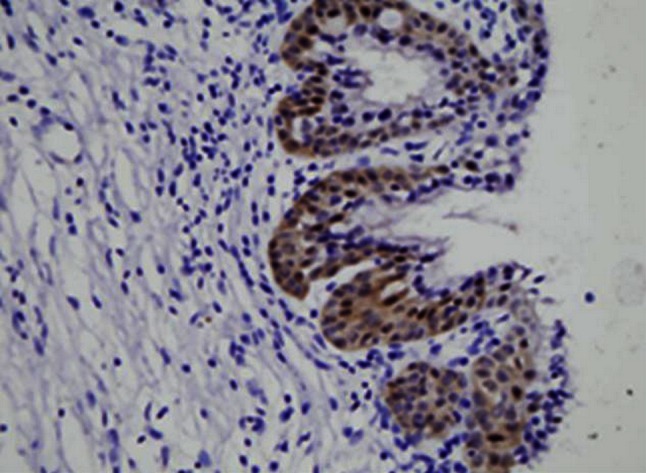
This figure shows maspin expression in squamous epithelium of normal cervix. Both cytoplasm and nucleus of squamous epithelial cells in normal cervix exhibited moderate to strong maspin expression (HE×400)

**Fig. 2:**
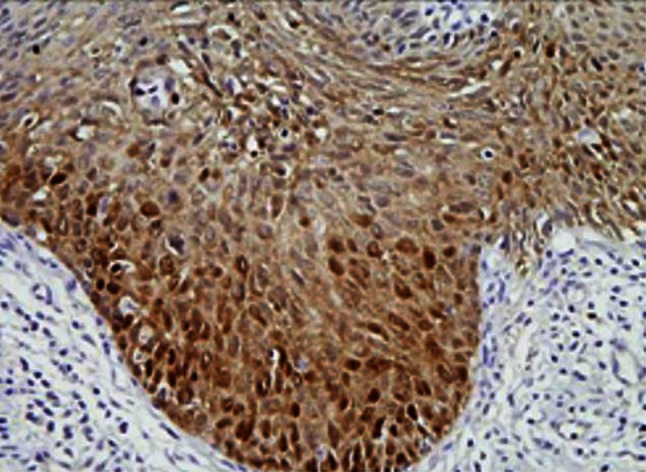
This figure shows maspin expression in CIN3. Both cytoplasm and nucleus of squamous epithelial cells in CIN3 exhibited moderate to strong maspin expression (HE×400)

**Fig. 3:**
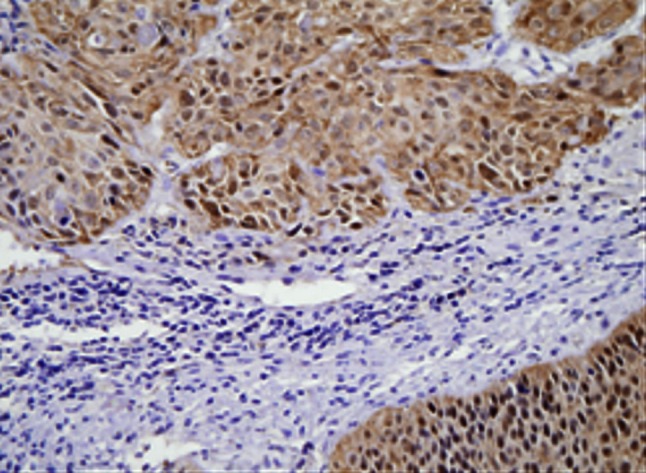
This figure shows maspin expression in SCC stage Ib. Both cytoplasm and nucleus of cells in SCC stage Ib showed obviously weaker staining of maspin than those of normal cervix and CIN3

**Fig. 4:**
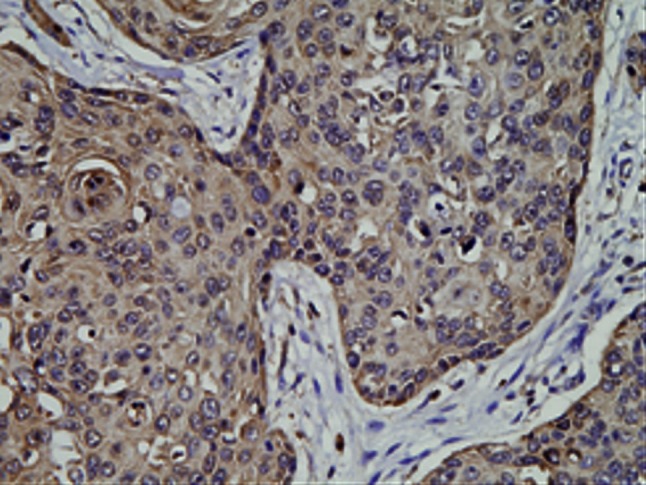
This figure shows maspin expression in SCC stage II. Cytoplasm in cells of SCC stage II exhibited moderate expression while nucleus of it showed weak maspin expression

**Fig. 5:**
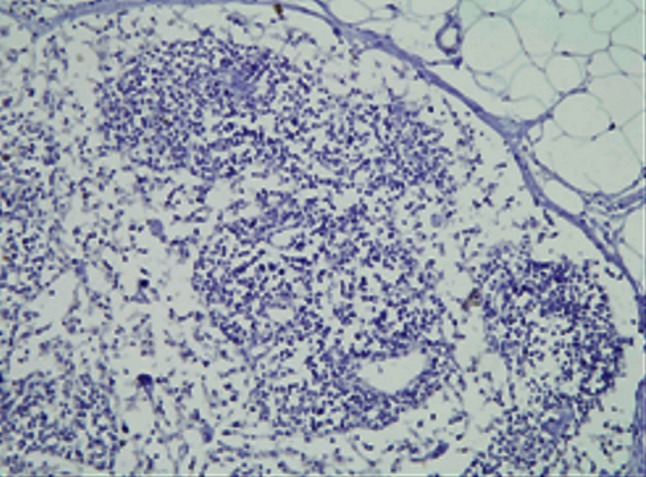
This figure shows maspin expression in lymph node free of tumor metastasis. No maspin  expressed in lymph node free of tumor metastasis

**Fig. 6:**
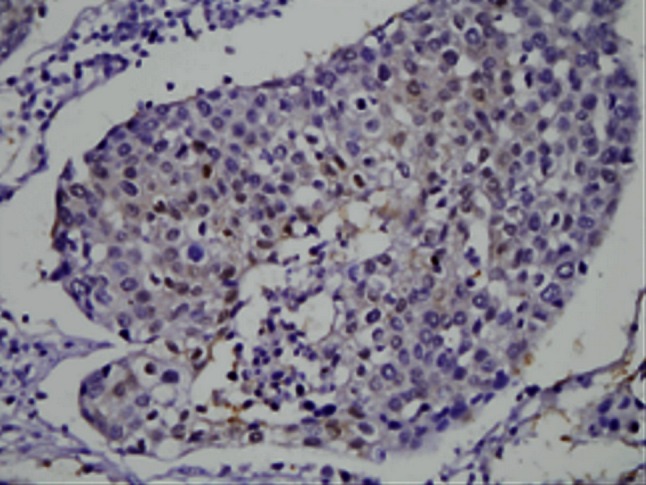
This figure shows maspin expression in tumor emboli of lymph nodes. Both cytoplasm and nucleus showed extremely weak maspin expression in tumor emboli of lymph nodes

Maspin expression was detected in both cytoplasm and nucleus of squamous epithelium cells of normal cervix, CIN3 and SCC of the uterine cervix while no maspin expressed in the lymph nodes free of tumor metastasis. Cytoplasm and nucleus of squamous epithelial cells in normal cervix and CIN3 exhibited moderate to strong maspin expression while SCC showed obviously weaker staining for maspin (*P* < 0.05) and tumor emboli in lymph nodes was the weakest (*P* < 0.05) (Figs. 1, 2, 3, 4, 5, 6).

Squamous cell carcinoma stage Ib cytoplasmic maspin showed no significant difference with SCC stage II (*P* > 0.05), but weaker than normal cervix and CIN3 (*P* < 0.05). In cytoplasm of tumor emboli in lymph nodes, maspin expression was obviously weaker than that of SCC stage II (*P* < 0.05) (Table 2). Compared with normal cervix, nuclear maspin expression of CIN3 was little up-regulated whereas that of SCC stage Ib was declined (*P* > 0.05) which was significantly weaker than CIN3 (*P* < 0.05). SCC stage II nuclear maspin was obviously weaker than SCC stage Ib (*P* < 0.05) but showed no statistical significance with tumor emboli in lymph nodes (*P* > 0.05) probably due to the number of cases with tumor emboli in lymph nodes (Table 3).

**Table 2:** Comparison of cytoplasmic maspin expression in different study groups

Compared groups A and B		Sample size		Mean rank of two groups		Significance level
		*n*_A_	*n*_B_	R_A_	R_B_	
1	−2^a^	13	15	83.92	78.80	
1	−3	13	15	83.92	55.60	*
1	−4	13	47	83.92	44.48	*
1	−5	13	13	83.92	12.19	*
2	−3	15	15	78.80	55.60	*
2	−4	15	47	78.80	44.48	*
2	−5	15	13	78.80	12.19	*
3	−4	15	47	55.60	44.48	
3	−5	15	13	55.60	12.19	*
4	−5	47	13	44.48	12.19	*

Comparison of cytoplasmic maspin expressions between any two groups using the Kruskal-Wallis rank sum test discovered SCC stage Ib cytoplasmic maspin showed no significant difference with SCC stage II but weaker than normal cervix and CIN3. Maspin expression in cytoplasm of tumor emboli in lymph nodes was obviously weaker than SCC stage II

* indicates *P* < 0.05

^a^Study groups: 1, normal cervix; 2, CIN3; 3, SCC stage Ib; 4, SCC stage II; 5, tumor emboli in lymph nodes

**Table 3:** Comparison of nuclear maspin expression in different study groups

Compared group A and B		Sample size		Mean rank of two groups		Significance level
		*n*_A_	*n*_B_	R_A_	R_B_	
**1**	**−2** ^a^	13	15	81.57	90.36	
**1**	**−3**	13	15	81.57	63.60	
**1**	**−4**	13	47	81.57	35.25	*
**1**	**−5**	13	13	81.57	25.30	*
**2**	**−3**	15	15	90.36	63.60	*
**2**	**−4**	15	47	90.36	35.25	*
**2**	**−5**	15	13	90.36	25.30	*
**3**	**−4**	15	47	63.60	35.25	*
**3**	**−5**	15	13	63.60	25.30	*
**4**	**−5**	47	13	35.25	25.30	

Comparison of nuclear maspin expression between any two groups using the Kruskal-Wallis rank sum test discovered nuclear maspin expression of CIN3 was little up-regulated than normal cervix and then declined in SCC stage Ib. SCC stage II nuclear maspin was obviously weaker than SCC stage Ib but showed no statistical significance with tumor emboli in lymph nodes

* indicates *P* < 0.05

^a^Study groups: 1, normal cervix; 2, CIN3; 3, SCC stage Ib; 4, SCC stage II; 5, tumor emboli in lymph nodes

### Podoplanin expression in SCC

**Fig. 7:**
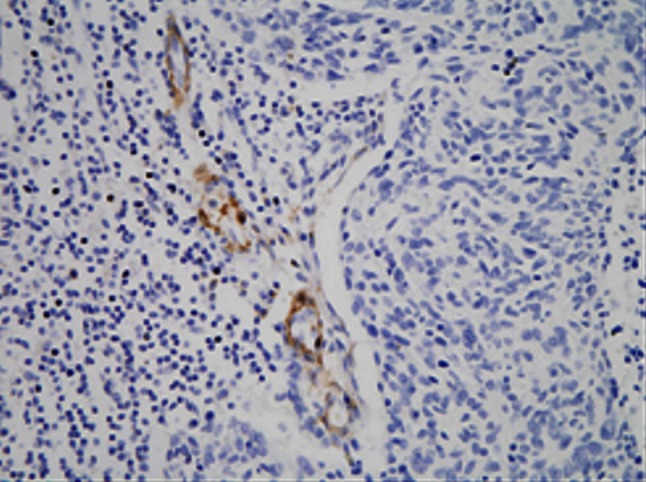
This figure shows lymphatic microvessels around tumor tissue of cervical cancer. Lymphatic microvessels stained with podoplanin in SCC stage Ib mostly located in the area around the tumor

**Fig. 8:**
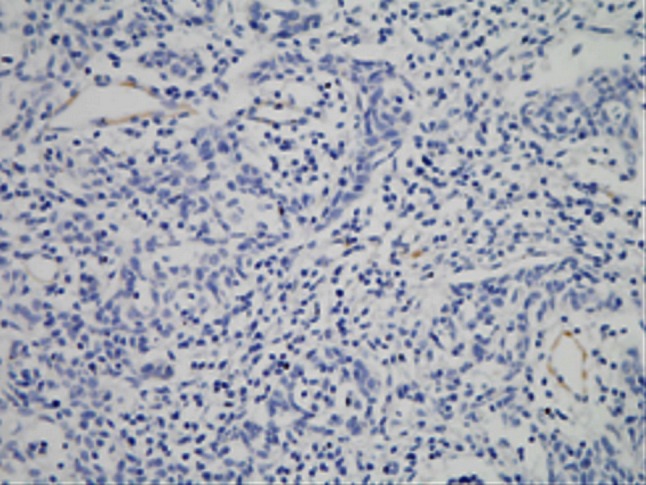
This figure shows lymphatic microvessels stained with podoplanin were rare or even absent in the internal parts of tumor in SCC stage II

Positive expression of podoplanin was deposition of yellow or brown particles in cytoplasm of endothelial cells of lymphatic vessels. The “hot spot” of lymphatic microvessels were mostly located in tumor interstitial tissues and periphery but rare or even absent in the internal parts of tumor (Figs. 7, 8). LMVD in SCC stage Ib and SCC stage II were 4.47 ± 1.73 and 6.26 ± 1.51 respectively which were significantly different (*P* < 0.05).

### Relationship among maspin expression, clinical and pathologic characteristics of SCC of the cervix and LMVD

It is showed in logistic regression analysis that nuclear maspin expression related to clinical stage of cervical cancer and lymphatic metastasis (*P* < 0.01, *P* < 0.05, respectively) (Table 4), while cytoplasmic maspin expression only related to the latter (*P* < 0.05) (Table 5) without association with clinical stage, invasive depth and histological grade (*P* > 0.05). LMVD related to clinical stage, lymphatic metastasis and histological grade (*P* < 0.005, *P* < 0.05, *P* < 0.05) but did not associate with invasive depth (*P* > 0.05) (Table 6). It is showed in Spearman rank correlation analysis that nuclear maspin expression was negative related to clinical stage and LMVD (*P* < 0.001, *P* < 0.005, respectively) whereas cytoplasmic maspin expression was only negative related to LMVD (*P* < 0.005) having no association with clinical stage of SCC (*P* > 0.05). LMVD was positive related to clinical stage (*P* < 0.01) (Tables 7, 8).

**Table 4:** Logistic regression analysis of nuclear maspin expression and clinical/pathologic characteristics of cervical cancer

Clinical**/**pathologic parameter	Degree of freedom	Estimate value of regression coefficient	Standard error	Wald Chi-square value	*P* value
Clinical stage	1	2.4459	0.6299	15.0785	0.0091
Invasive depth	1	−0.2774	0.5772	0.2310	0.6308
Lymphatic metastasis	1	2.3894	0.6636	12.9651	0.0183
Histological grade	1	0.6547	0.5473	1.4308	0.2316

It is showed in logistic regression analysis that nuclear maspin expression related to clinical stage of cervical cancer and lymphatic metastasis

**Table 5:** Logistic regression analysis of cytoplasmic maspin expression and clinical/pathologic characteristics of cervical cancer

Clinical**/**pathologic characteristics	Degree of freedom	Estimate value of regression coefficient	Standard error	Wald Chi-square-value	*P* value
Clinical stage	1	0.6617	0.5671	1.3614	0.2433
Invasive depth	1	−0.0608	0.5821	0.0109	0.9169
Lymphatic metastasis	1	2.2852	0.6448	12.5621	0.0104
Histological grade	1	−0.0194	0.5361	0.0013	0.9711

Logistic regression analysis shows that cytoplasmic maspin expression only related to lymphatic metastasis

**Table 6:** Logistic regression analysis of LMVD and clinical/pathologic characteristics of cervical cancer

Clinical**/**pathologic parameter	Degree of freedom	Estimate value of regression coefficient	Standard error	Wald Chi-square value	*P* value
Clinical stage	1	−1.7374	0.5909	8.6439	0.0033
Invasive depth	1	−0.3552	0.5765	0.3797	0.5378
Lymphatic metastasis	1	−2.4775	0.6536	14.3663	0.0127
Histological grade	1	−1.1024	0.5453	4.0874	0.0432

Logistic regression analysis shows that LMVD related to clinical stage, lymphatic metastasis and histological grade

**Table 7:** Spearman rank correlation analysis of nuclear maspin expression of SCC, clinical stage of cervical cancer and LMVD

	Maspin expression in nucleus	LMVD	Clinical stage
Maspin expression in nucleus	1	−0.8213 <0.0019	−0.4919 <0.0001
LMVD	−0.8213 <0.0019	1	0.4214 0.0026
Clinical stage	−0.4919 <0.0001	0.4214 0.0026	1

The first line of each cell is the correlation coefficient and the second line is the *P* value

Spearman rank correlation analysis shows that nuclear maspin expression was negatively related to clinical stage and LMVD

**Table 8:** Spearman rank correlation analysis of cytoplasmic maspin expression of SCC, clinical stage of cervical cancer and LMVD

	Maspin expression in cytoplasm	LMVD	Clinical stage
Maspin expression in cytoplasm	1	−0.5582 <0.0011	−0.1986 0.1218
LMVD	−0.5582 <0.0011	1	0.4214 0.0086
Clinical stage	−0.1986 0.1218	0.4214 0.0086	1

The first line of each cell is the correlation coefficient and the second line is the *P* value

Spearman rank correlation analysis shows that cytoplasmic maspin expression was negatively related to LMVD, which was positively related to clinical stage

## Discussion

Carcinoma of the uterine cervix was one of the leading ranks of malignant disease in female genital tract. Worldwide, cervical cancer is second only to breast cancer as the most common cancer in terms of both incidence and mortality rate. As we know, in cervical cancer, metastasis through lymphatic vessels is the most common way to spread out and is also a cardinal factor affecting prognosis of patients. Birner et al. [26] discovered there had already been lymphatic vessels involvement in 35.7 % of cases in early stages of cervical cancer and the disease-free survival rate for 5 years was obviously lower in the group with lymphatic vessels involvement than the group without it and there are lymph nodes metastasis in 65.7 % of patients with lymphatic vessels involvement indicating that there is a correlation between lymphatic vessels involvement and lymph nodes metastasis. Therefore, close attention has recently been paid to both the effect of lymphatic microvessels on tumor lymphatic metastasis and the regulation of lymphangiogenesis. Any tool allows predicting the potential for progression and metastasis in patients with cervical cancer may be clinically valuable.

Maspin, a serine protease inhibitor, was originally identified in normal human breast epithelial and myoepithelial cell lines [2]. It has been widely proved that maspin expressed in many kinds of human tissues and organs, such as breast, prostate, gastrointestinal tract, endothelium of the uterine, and trophocyte of the placenta. After Zou et al. [4] first reported that maspin expression obviously descended in invasive breast carcinoma, the same expression pattern had been discovered in many other kinds of tumors such as prostate cancer, renal cell carcinoma, oral cavity squamous cancer, gastric carcinoma and colonic cancer [3, 5, 7, 27]. However, not all kinds of tumors support the above pattern. Contrasted with the early research, maspin expression was weak or even absent in normal ovarian tissue but it strongly expressed in ovarian cancer [12]. Other studies showed that maspin expression was positive related to tumor size and histological grade but negative related to relapse free survival and total survival span [10, 16–18]. Some people even took the viewpoint that maspin expression only strengthened in invasive breast cancer of high histological grade and maspin over expression means high risk of death for patients without lymphatic metastasis [28]. These paradoxical results of different studies is intriguing and most probably reflects the variation in pathogenesis of tumors in different organs as well as sufficiently demonstrated the complexity of maspin expression and its effect on tumor progression and metastasis. Although the mechanism of biological function of maspin is still unclear, it has been demonstrated that maspin inhibits tumor cell motility, invasion and metastasis as well as tumor angiogenesis [29]. These findings support the tumor suppression role of maspin in tumor progression. But only a few studies have mentioned maspin expression in the cervix.

Furthermore, it has been observed that even deeper significance may exist in subcellular localization of maspin. Maspin located mostly in cytoplasm in invasive tumor while it expressed stronger in nucleus in benign or mild malignant tumors [30, 31]. Subcellular location of maspin expression may predict the different clinical types of tumors and prognosis of patients [4, 18–20].

In our study, maspin expression is detected in both cytoplasm and nucleus of normal cervix, CIN3, SCC and tumor emboli in lymph nodes which obviously decreased in the latter two groups (*P* < 0.05) and showed the weakest expression or even absence in tumor emboli in lymph nodes. According to our result, maspin expression in cervical cancer just like that in breast and prostate cancer and support its tumor suppressor role.

Both cytoplasmic and nuclear maspin expressions of SCC stage Ib were obviously weaker than normal cervix and CIN3 (*P* < 0.05). Diminished cytoplasmic maspin expression is not identified in CIN3 compared with normal squamous epithelium while nuclear maspin expression in CIN3 was a little upregulated than both normal cervix and SCC. The upregulation of nuclear maspin expression in CIN3 may be a stress reaction to cancerigenic factors in vivo or vitro or to an oncogene that will be or has already been activated. As maspin has been proved a regulation target of gene p53 [32, 33] and maspin expression is either negative related to p53 expression or increased with the step-up of abnormal expression of it [34], maspin may be the replacement or supplement for insufficient or even lost function of anti-oncogene p53 as a secondary tumor suppressor gene. Increased nuclear maspin expression in CIN3 could be the earliest change happens when CIN3 or carcinoma in situ will soon progress to invasive cancer. Xu Cet al. [1] had discovered that maspin expression in cytoplasm gradually descended in CIN3, micro-invasive and invasive squamous cell carcinoma of the uterine cervix and the level of cytoplasmic maspin expression in CIN3 adjacent to invasive cancer is lower than that in CIN3 without invasive carcinoma, but the nuclear maspin expression has not been mentioned. Combined with our study, we think it is the early event of tumor invasion that maspin increased in nucleus while diminished in cytoplasm in CIN3 and maspin plays a role in disease progression from CIN3 to invasive squamous cervical carcinoma.

Squamous cell carcinoma stage II nuclear maspin expression was obviously weaker than SCC stage Ib (*P* < 0.05) although cytoplasmic maspin expression showed no significant difference between the two groups (*P* > 0.05). SCC stage II cytoplasmic maspin expression was stronger than that of tumor emboli in lymph nodes (*P* < 0.05) while nuclear maspin expression was extremely weak in both groups. If take the nuclear maspin expression level of tumor emboli in metastatic lymph nodes as the “baseline” and draw the curve of nuclear maspin expression from normal cervix to metastatic carcinoma, it will show a peak in CIN3 and then gradually dropped which indicate that nuclear maspin first react to oncogenic factors and then direct the change of maspin in cytoplasm. Extremely weak SCC stage II nuclear maspin expression demonstrated the exhaustion of tumor suppression effect of maspin and increased potential for local invasive and lymphatic metastasis in spite of the cytoplasmic maspin expression was still maintained. Nuclear maspin expression of both SCC stage II and tumor emboli in metastatic lymph nodes were extremely weak and existed no statistical significance not only support the above viewpoint but also demonstrated the possibility that tumor cells in metastatic lymph nodes come from the primary cancer sites of SCC.

As we know, microvasculature plays an important role in the process of tumor invasion and metastasis. In cervical cancer, metastasis through lymphatic vessels is the most common way to spread out. Schoppmann et al. [35] have discovered increased LMVD and lymphatic vessels involvement had a relationship with axillary lymph nodes metastasis when they used podoplanin to label lymphatic microvessels in invasive breast carcinoma. In present research, after labeling with podoplanin, the lymphatic microvessels in cervical cancer were discovered mostly in interstitial tissue and periphery of the tumor but rare or even absent in the tumor tissue. LMVD in SCC stage Ib is lower than that in SCC stage II with statistical significance (*P* < 0.05) demonstrating LMVD gradually increased with the progression of SCC of cervix.

Some researchers have demonstrated that maspin expression was negative related to tumor stage, histological grade and depth of invasion [3–7]. Downregulated or absent of maspin expression may signify the increased potential for tumor metastasis and poor prognosis [6, 7, 9]. Our study shows that nuclear maspin expression had inverse correlation with clinical stage (*P* < 0.05) and metastasis of lymph nodes (*P* < 0.05), that is, the lower the nuclear maspin expression, the later the clinical stage of cervical cancer, and the metastasis of lymph nodes is more likely to happen. As we know, lymphatic metastasis is still an important factor for prognosis; our research could indicate that nuclear maspin expression was significantly related to tumor progression and metastasis of local lymph nodes and has further significance in predicting pelvic lymph nodes metastasis and prognosis of patients.

Invasive depth is another important factor for prognosis of patients, although our research did not show any statistical significance in nuclear maspin expression between the two groups that invasive depth is less than or equal to or more than 1/2 of the whole layer, it disclosed from another point that change of nuclear maspin expression is the early event in tumor progression and metastasis. And now, the clinical dividing of less than or equal to and more than 1/2 of the whole layer in invasive depth may be too rough to detect the molecular biological change that happened in cells of early stage in tumor progression. The differentiation grade is also a factor affecting prognosis of patients. Generally speaking, the poorer the differentiation grade is, the worse the malignant grade and prognosis is. Our research did not show there is a relationship between nuclear maspin expression and cell differentiation could be explained as neither maspin expression nor cell differentiation grade is an independent factor that will affect the progression of cervical cancer.

It has been showed in most studies that the formation of newborn lymphatic vessels promoting the lymphatic metastasis of tumors and the higher the LMVD is, the easier the tumor cells go into the lymphatic system [36, 37]. Some studies also suggested that the area around tumor is more suitable for development of lymphatic microvessels and it is more likely lymphatic microvessels of this area participate in the metastasis and spreading of tumor cells [38]. It has also been proved in our research that LMVD increased in periphery of the tumor while obviously diminished intratumorally. LMVD was obviously higher in SCC stage II than in SCC stage Ib (*P* < 0.05) and the increase of LMVD was obviously related to late clinical stage, lymph nodes metastasis and poor differentiation grade (*P* < 0.05). So local lymphatic microvessels may develop important effect on lymphatic metastasis in cervical cancer.

Although maspin has been proved to be related with the decrease of density of tumor capillary in both breast and colonic cancer [39, 40] and can inhibit the formation of blood vessels in tumors [31, 41], few reports have mentioned the relationship between maspin expression and lymphatic microvessels until now. It is generally thought, primitive vein is the common origin of both blood and lymphatic vessels. They differentiate under disparate regulation mechanisms. The homology is also manifested in fact that they are both promoted by VEGF and inhibited by classic angiostatin such as inhibin and platelet factor IV [42]. But does maspin, which has the obvious inhibitory effect on angiogenesis do the same on lymphangiogenesis? The result of our study has demonstrated that maspin expression both in cytoplasm and nucleus was obviously negative related to LMVD (*P* < 0.05) from which we could predict that maspin plays an inverse impact on lymphangiogenesis in the progression of squamous cell carcinoma of the uterine cervix and in a certain degree, it delays or even holds the metastasis of local lymph nodes. But details about it still remain unclear and further evidence is needed for making a decision.

In conclusion, subcellular location of maspin expression could be a potentially useful marker to identify the progression and prognosis of patients in cervical cancer since maspin expression correlated with clinical stage and lymphatic metastasis of the tumor. Maspin expression in both cytoplasm and nucleus exists a negative relationship with LMVD in cervical cancer support a role for maspin in lymphangiogenesis and lymphatic metastasis.

## Abbreviations

CIN3: Cervical intraepithelial neoplasia grade 3
LMVD: Density of lymphatic microvessels
SCC: Squamous cell carcinoma
